# Influence of Parental Attitudes on Formation of Psychological Resilience and Adherence to Medical Regime in Adolescents after Liver or Renal Transplantation

**DOI:** 10.3390/children8080619

**Published:** 2021-07-22

**Authors:** Marta Biernacka, Anna Jakubowska-Winecka, Piotr Kaliciński

**Affiliations:** 1 Department of Health Psychology, The Children’s Memorial Health Institute, 04-730 Warsaw, Poland; A.Jakubowska-Winecka@ipczd.pl; 2 Department of Pediatric Surgery & Organ Transplantation, The Children’s Memorial Health Institute, 04-730 Warsaw, Poland; p.kalicinski@ipczd.pl

**Keywords:** resilience, parental attitudes, adherence in therapeutic process

## Abstract

Identifying the causes of poor disease control and medication non-adherence is indispensable so that patients can benefit from treatment. The aim of our study was to determine the relationship between parental attitudes, the development of psychological resilience, and systematic medication adherence in a group of adolescents after kidney and liver transplantation. The analysis included the results obtained from 96 families. A total of 52 patients after kidney transplantation and 44 patients after liver transplantation, aged 12–18 years and their parents were examined. The types of parental attitudes were assessed using the Parental Attitude Scale. The patient’s resilience was determined with the Resiliency Assessment Scale. The MMAS-8 was used to assess the regularity of medication-taking behavior. A total of 61% of the patients in the study group displayed high levels of psychological resilience. The analyses showed a positive correlation between resilience and the systematic taking of medication by the patients. Moreover, it was found that the analyzed link between psychological resilience on the degree of the regularity of medication intake was enhanced by a specific type of parental attitude. The obtained results confirm the importance of psychological resources in developing better disease control. The relationship between the type of parental attitudes and medication adherence indicates the need to take into account the family context during the child’s treatment.

## 1. Introduction

A particularly important area of research is the issue of cooperation in the course of treatment. In chronic diseases, regular medication intake constitutes the basis of therapy effectiveness and reduces the risk of complications and relapse [1]. Additionally, it allows one to gain psychosocial benefits from treatment and to avoid negative psychological consequences, i.e., the most frequent anxiety disorders and depression, which significantly reduce the ability to cope with the situation of being ill and being treated. In the group of adolescents, a conflict between the requirements of treatment and the developmental tasks, so typical of adolescence, is clearly visible: the achievement of autonomy, the maturation of cognitive competences favoring the critical assessment of the surrounding world, the search for one’s own life path and the negation of authorities [2]. Hence, many patients within the indicated age group do not accept the limitations imposed by their own disease and treatment restrictions; as a result of which, the selective use or complete discontinuation of pharmacotherapy can be observed.

Against this background, it is important to undertake research aimed at identifying potential factors that support adolescent patients’ adaptation processes and cooperation in the course of treatment. On the other hand, it is essential to identify disruptive factors in order to adjust them and intervene accordingly.

Psychological resilience is mentioned among the various psychological resources facilitating beneficial adaptation to illness. Resilience is revealed in the context of coping with negative life events and difficulties [3]. In this sense, resilience can be viewed as an indicator of mental strength. Thanks to it, despite adversities, a person can develop and maintain mental health. Resilience is especially important in the current pandemic situation, where societies are feeling, and struggling with, the psychological effects of the crisis. Dealing with problems requires the use of one’s own psychological resources as well as the ability to exploit the properties of the environment, to flexibly adapt strategies to situations that arise, and to draw on knowledge, social relations and emotional competence. Veer et al. [4], in their work on resilience factors, list several of them, attributing to them the function of psychological resources influencing one’s ability to constructively cope with the effects of a pandemic. In the aforementioned studies, a positive valuing style, perceived social support, and the ability to recover from stress constitute a kind of buffer, reducing the impact of stress. These resilience factors seem to be equally important for successful adaptation to stress in the context of chronic illness and its treatment. When it comes to a chronic illness, psychological resilience is a resource that promotes adaptation to the circumstances of the illness and the limitations generated by it [5]. Individuals characterized by high levels of resilience have an easier ability to initiate remedial action and are more persistent in the implementation of the undertaken activity in connection with the manifested higher sense of self-esteem and effectiveness. The patient, thanks to personal beliefs about his/her strength and abilities, focuses on active actions rather than obstacles, which prevents the occurrence of negative emotional states that preclude good cooperation in the process of treatment. When faced with life’s adversities, people with low resilience respond with severe stress, rigid behavior, and negative emotions. Wifield [6] indicates that resilience is associated with social support received from parents and teachers. In our study, we link resilience to parental attitudes.

Undoubtedly, parental attitudes influence the development of psychosocial competences and resources in children. The level of parental emotional commitment and the degree of autonomy granted to the child allow him or her to develop the level of resilience needed to master environmental challenges. The work of Goldschmidt et al. [7], on the other hand, highlights the importance of the role of parents in the context of children’s school achievement. The study of cognitive functioning after transplantation is becoming increasingly common. The authors of this study demonstrated that a variable such as parental educational status significantly correlates with children’s school achievement, but this is not the case for the group of children after liver transplantation. It seems likely that the chronicity of the disease and numerous hospitalizations limit the patients’ level of adequate cognitive stimulation, translating into poorer cognitive development. This is a possible explanation, but not the only one. The situation of patients with organ disease is a situation considered in terms of stress. A child experiencing numerous stressful situations related to the disease, deprived of normal environmental interactions, may not have developed ways of coping with strong negative emotions. Chronic experience of stress is a risk factor for later cognitive deficits. In this sense, resilience becomes a protective factor, taking on a broader meaning to counteract patients’ cognitive deficits.

The research based on a meta-analysis [8] highlights the importance of family functioning as an important factor contributing to the growth of desirable behaviors and good disease control. In our study, an assumption was made that higher levels of resilience in the subjects under investigation who underwent organ transplantation would have an adaptive function that would be reflected in the regularity of medication intake. We also wondered whether the indicated relationship was moderated by parental attitudes. The aim of the presented study was to assess the degree of adaptation of adolescents after organ transplantation to the context of chronic disease. We used the degree of regularity of medication intake as an indicator of adaptation. We examined which of the patients’ psychological resources were important for shaping and optimizing the cooperation in the process of treatment.

## 2. Materials and Methods

### 2.1. Study Design

The present cross-sectional study is a part of a larger work on the psychological determinants of the cooperation of children and adolescents with a chronic illness. The patients were invited to participate in the study during hospitalization or follow-up visit in the clinic. The participation in the study required the patient’s and his or her parent’s informed consent. The study was conducted by a clinical psychologist, with the approval of the bioethics committee.

### 2.2. Patients Characteristic

A total of 96 patients aged 12 to 18 years participated in the study (M = 14.70 SD = 1.836). Among those participating in the study, 52 patients were kidney transplant patients (24 girls and 28 boys) and 44 liver transplant patients (25 girls and 19 boys). Apart from patients, the study also included their parents. One parent per patient participated in this study. Mainly the mothers of the patients participated in the study (77 females and 19 males, *p* < 0.05). The mothers of the patients represented 80.2% of the total parental group. The time post-transplantation varied and ranged from 1 to 17 years: for kidney recipients, it was 5 years (2–12) (median and range), and 9 years (2–17) for liver recipients.

### 2.3. Measures

The Parental Attitudes Scale (pol. SPR) by Plopa [9] was used in this study. The aforementioned tool contains 50 statements, with a five-point Likert scale. It facilitates an assessment of parental attitudes in the subjective perception of the parent. The scale distinguishes 5 types of parental attitudes: 1: Acceptance–rejection (spr_acc): the parent’s attentiveness and sensitivity to the needs and behaviors of the child. They demonstrate a high degree of emotional involvement vs. being emotionally cool, distant, and mentally indifferent in interactions. 2: Excessively demanding (spr_dem): the parent is critical of the child’s accomplishments, makes most decisions for the child, and accepts only those choices that are consistent with his/her views. 3: Autonomy (spr_aut): the parent respects the child’s privacy and accepts the child’s views even if they are not fully shared. 4: Inconsequent (spr_inc): the parent’s relationship with the child is volatile, depending on mood, and is unpredictable. The parent’s instability causes the child’s distancing and timidity; and 5: excessively protecting (spr_prot): the parent treats the child as needing constant care and attention and limits the child’s efforts and freedom. The scores are calculated separately for each scale, and they range from 10–50 points. Using the results obtained, a predominant type of parental attitude can be identified.

The level of psychological resilience in patients was assessed with the Resiliency Assessment Scale for Children and Adolescents (SPP-18) by Ogińska-Bulik and Juczyński [10]. The scale contains 18 statements with a five-point Likert scale. The SPP reflects to the child’s attitude towards the ability to handle a difficult situation. The tool allows for the determination of personal resilience at the level of the total score (SPP) as well as the four factors it includes, i.e., (1) an optimistic attitude and energy, (2) persistence and determination in action, (3) a sense of humor and openness to new experiences and (4) personal competence and tolerance of negative effects. The higher the score, the higher the intensity of resilience. The values in the SPP test range from 0 to 72 points. The variables used in the study are continuous variables. However, the terms low and high represent -1SD for a low level of a trait and +1SD for a high level of a trait.

The Morisky Medication Adherence Scale MMAS-8 tool was used to estimate the regularity of medication intake, with permission given by the author [11]. It is a self-report scale containing eight questions on the issue of regular medication adherence. The author of the scale provided yes or no answers for 7 questions. The final question requires patients to select one of five possible answers. With the help of the scale, it is possible to determine the degree of the patient’s cooperation in the process of treatment: the higher the score on the MMAS-8, the greater the regularity of medication intake. The scale distinguishes three levels: low (<6); medium (6 < 8) and high (=8).

### 2.4. Statistical Analysis

The level of each variable in the study group was characterized using descriptive statistics (mean and standard deviation). A Student’s t-test was used to determine the differences between the level of the studied variables and the values in the general population. For the calculations, the values included in the manual for SPP-18 (2011) were used, which were matched in terms of the characteristics of the group of subjects participating in our study (sex and age concordance). A Student’s t-test was also conducted to determine whether individuals with different disease types differed in the level of the study variables. The next step of the analysis was to perform r-Pearson correlation between the studied variables. Since the study variables were correlated with each other, the next step of analysis was to conduct multivariate regression analysis. The regression analysis allowed for the identification of psychological predictors of medication adherence regularity. Within the regression analysis, a moderation analysis was conducted, where the moderator was parental attitudes, the independent variable was resilience, and the dependent variable was medication adherence regularity. Data included in the regression model were precentered relative to the median. The data presented in the graphs are based on the points determined by the standard deviation values, where a low level of a trait represents -1SD and +1SD refers to high level of a trait. The distribution of variables was tested using the Kolmogorov–Smirnov test. PASW 21 (SPSS Inc, Chicago, IL, USA) was used for statistical analysis.

## 3. Results

The mean score obtained by the adolescents in question in the MMAS-8 questionnaire was 6.83, which meant that the regularity of taking medication was at an average level. The average resilience score in the total score for the whole study group of patients was M = 52.67; SD = 9.284. Both the mean of the general resilience score and its four factors were significantly higher than in the normalization group (*p* = 0.001). Within the whole group, more than half of the subjects under analysis (61.5%) obtained a high level of resilience, 34.4% of the examined adolescents a medium level, and 3.1% a low level. Sex and age differentiated neither the intensity of resilience in the total score nor the individual components and the degree of medication intake. The group of parents displaying an attitude of acceptance was the largest group within the study group, which accounted for 44.3% of the respondents. These were followed by the autonomy attitude (37.91%), excessively protecting attitude (30.26), excessively demanding attitude (27.82%) and inconsequent attitude (20.84%).

The Student’s t-test was used to determine if the level of the study variables differed between the groups. The results are shown in Table 1.

The above results do not indicate the existence of differences within the examined variables between adolescents after kidney transplantation and after liver transplantation. It means that the examined adolescents constitute a homogeneous group in this respect, and therefore, further analyses will be carried out for the whole group.

The r-Pearson correlation tested whether, as expected, psychological resilience would be positively correlated with the level of medication regularity in our group of adolescents. It was also determined if and which parental attitudes are associated with the dependent variable. The results can be seen in Table 2.

The correlation coefficients presented in Table 2 indicate significant but moderate correlations between the analyzed variables. The overall level of resilience is significantly associated with the regularity of medication intake: r = 0.256, *p* = 0.012. Patients displaying higher levels of psychological resilience more regularly follow medical recommendations concerning taking medication. A significant association was also found for parental attitudes. The attitude described on the acceptance–rejection dimension, r = 0.225, *p* = 0.029, and the excessively protecting attitude, *p* = 0.271, *p* = 0.008, were significantly associated with regular medication adherence in children. Furthermore, it can be concluded from the above table that the psychological variables analyzed in our study are correlated with each other. Therefore, the next step of the analysis was to perform multivariate regression analysis. The regression analysis provided an answer to the question as to which of the study variables was the best predictor of medication adherence regularity. The significance of the interaction between the psychological variables studied was also established. The results are presented in Table 3.

**Figure 1:**
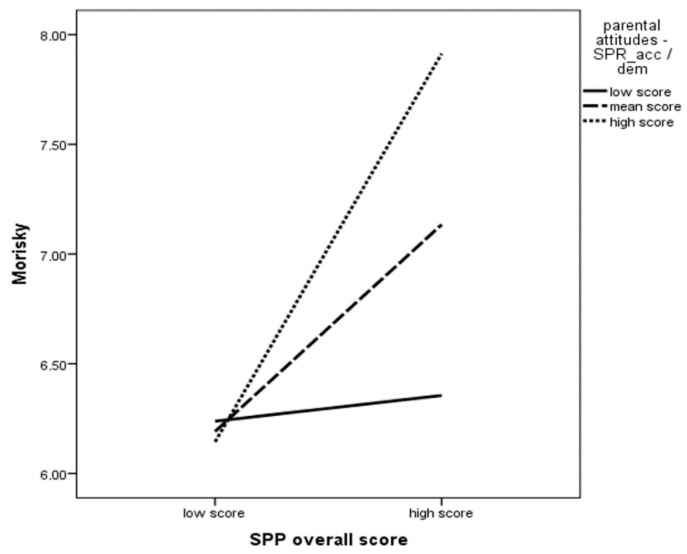
SPP and parental attitudes interaction effect on medical adherence (Morisky overall score).

Table 3 shows that patient resilience plays a crucial role in predicting medication regularity F (1.94) = 6.502, *p* = 0.12, and its contribution is clarified by approximately 6.5%. In the next step of the analysis, individual dimensions of parental attitudes were additionally taken into account, which also turned out to be statistically significant: F (6.94) please keep the comma in F = 2.622, *p* = 0.22, where for the whole equation, R^2^ = 0.152. It means that we can predict about 15% of the regularity of taking medication on the basis of the studied variables. However, the increment of the variance of the dependent variable being explained with respect to the previous model was not statistically significant: *p* = 0.123. The next step of the analysis showed that the regularity of taking medication could also be explained by the type of parental attitude characterized by excessive protectiveness: β = 0.281, *p* = 0.043. The patients whose parents were more caring and protective showed regularity in taking medication. The mentioned variables, i.e., excessively protective attitude and resilience, explained another part of the variance of the dependent variable, i.e., the regularity of medication intake. The last regression model, additionally including the interaction between resilience level and parental attitudes, was also statistically significant: F (11.94) = 2.545, *p* = 0.08, where for the whole equation, R^2^ = 0.252 and the change in R^2^ was statistically significant: *p* = 0.05. Of all the interactions included in the aforementioned equation, the interaction between the level of resilience and parental accepting attitude was found to be statistically significant: β = 0.412, *p* = 0.047. It means that the impact of resilience on the regularity of medication intake is greater the greater the level of acceptance attitude. Thus, there is a synergistic effect. The results are presented in Figure 1.

## 4. Discussion

Chronic illness involves difficult experiences and is associated with stress, which is a risk factor for psychosocial development and interference with the child’s daily functioning. For this reason, research is being conducted into factors that buffer stress and have a protective effect in difficult situations [12]. One of these is psychological resilience [13]. Evidence from the literature suggests that different types of difficult experiences can, due to their specific challenges and adaptive demands, result in developmental achievements in the form of extended resources and personal competences [14]. They can teach the flexible adaptation of coping strategies to the situations that arise, based on one’s own knowledge, the information obtained, social relationships and emotions. In the situation of chronically ill children, the living environment, the parents, the treatment team, and the school can contribute to this by supporting and providing a possibility to use the characteristics of the environment. Our analyses provided an interesting finding to support the above, namely that our patients achieve higher immunity compared to the general population. The daily lives of our patients differ from that of a healthy adolescent. We can assume that the experience of chronic illness and the support received from the environment have made our patients “resilient” and developed their adaptive competence. The construct of resilience is complex and multidimensional in nature. Therefore, another explanation for the above research result that comes to mind is the defense mechanisms that cause our subjects to need to present themselves as resilient. In this way, they can maintain psychosocial balance when experiencing the chronic stress of chronic illness. Both suppositions would require more in-depth analyses of, for example, patients’ psychosocial resources.

Resilience can be treated as psychological resilience, the ease of “recovery” with regard to confronting life’s problems, thereby facilitating positive adaptation to difficult situations. Positive adaptation to illness means having the personal competences needed to implement developmental tasks relevant to each stage. Cohler, Stott, Musick [15] point to the need for the development of psychological resilience in the prevention of mental and social problems. They point to the high costs that societies incur with regard to the treatment of patients. The authors of the present study focused on the role of resilience in adherence to medical recommendations. While reviewing research on the formation of psychological resilience in childhood, Luthar and Zigler [16] explained the complexity of the phenomenon: they presented the intertwining of life circumstances with the child’s psychosocial resources for the formation of resilience or vulnerability to stress, taking into account the life situation of the family.

The importance of family functioning in the process of coping with a child’s illness is pointed out by McCllean and Cohen [17]. Parents in situations of chronic illness transmit signals of distress to the child, which may weaken the child’s sense of security and inhibit the development of coping competences. In the present study, two groups of adolescents were investigated. The literature suggests that adherence to medical recommendations is low in the said age group. Fielding and Duff [18] in their paper discuss factors that support adherence to treatment protocols and risk factors, among which, they mention the age of adolescence. Their findings suggest that the younger the children, the higher the adherence to pharmacotherapy recommendations; however, when medical procedures are performed, it is more difficult to ensure the cooperation of young children. In addition, citing data from the literature, they report that children whose parents are more supportive, more flexible, less critical and adept at problem solving have fewer problems with regard to complying with medical recommendations. A conclusion can be drawn from the above-mentioned findings that not only the type of parental attitude but also the flexibility or resilience of the parent matter. In a slightly older study from 1993, Manne, Jacobsen, Gorfinkle et al. [19] analyzed the role of parental attitudes on adherence to cancer treatment requirements. Caregivers who exhibited a “supportive” parenting style were more responsive to their children, had a less restrictive approach to parenting and were more nurturing, cancelled fewer appointments, were more likely to arrive punctually for checkups and reported reactions to treatment with less delay.

The results of our study are largely consistent with the ones available in the literature. The regularity of medication intake in the groups of adolescents after kidney and liver transplantation was average. It is interesting to note that the two groups, despite differing in the type of disease and the degree of health and life risk due to graft loss, proved to be homogeneous in terms of the variables studied. In line with the literature, the level of resilience was found to be associated with the regularity of medication intake. A significant association was also found for parental attitudes. An acceptance attitude and an excessively protective attitude were statistically significantly associated with regular medication intake in children. The aforesaid result should be taken with great care because excessive protection is not a desirable parenting style, especially for adolescents, as it implies the assumption of control and responsibility for illness and treatment by caregivers. This, in turn, can create conflict and undermine the teen’s commitment to the treatment process.

An attitude of acceptance and recognition of autonomy seems most beneficial at any age. An accepting parental attitude when a child is ill is characterized by the parent’s emotional involvement, providing support and care. The parent focuses on the child, rather than on the illness and own needs. In contrast to the demanding attitude, for example, the parent does not expect the child to take control and responsibility for complying with medical recommendations: taking medication and complying with all treatment requirements. It also does not shift the care of the child to other people or institutions. Rather, it allows the child to gradually assume responsibility for the treatment process according to the child’s developmental capabilities. This attitude enables the child to acquire competence and adapt to the circumstances of a challenging chronic illness.

The results of our study showed an association of accepting attitude with resilience of the adolescents studied, which would mean that this style of parenting promotes the development of adaptive competence. Moreover, the above result is relevant to the regularity of medication intake. With adolescent patients’ adherence to medication consistently at an average level, the results of our study take on particular significance. We find that resilience, which is associated with adherence to health behaviors, may be enhanced by external factors, such as parental attitudes.

A variety of factors, not just those of the patient, may be responsible for failure in the patient’s treatment process. Interactions should consider the broader context of the situation. The consideration of parental attitudes in the child’s treatment process may be helpful, ultimately saving the patient from exacerbation. In addition, educating caregivers and increasing their awareness of the impact of their behavior on the attitudes and health behaviors of the patients themselves may increase the level of cooperation with the physician.

## 5. Conclusions

The results of the analysis confirm the significant role of psychological resilience in the process of positive adaptation to the disease and treatment in groups of adolescents after kidney and liver transplantation. It was found that the higher the level of resilience, the greater the regularity of taking necessary medication.

The obtained results also indicate the moderating role of certain types of parental attitudes in adherence to pharmacotherapy in adolescents.

The relationships revealed in the analysis of the results between the type of parental attitudes and psychological resilience and regularity of taking medication indicate synergistic relationships. In practice, this implies the need to educate caregivers about adolescents taking control and responsibility for treatment outcomes rather than parents.

## Figures and Tables

**Table 1:** Mean values of MMAS-8, resilience and parental attitudes depending on the type of disease.

	Disease	N	M	SD	t-Test	*p* Value (Two Tailed)
MMAS_8	LTx	44	6.69	1.222	−0.929	0.356
	KTx	51	6.94	1.36		
SPP_overall result	LTx	44	53.41	8.984	0.715	0.476
	KTx	51	52.04	9.579		
spr_acc	LTx	44	44.2	4.835	−0.155	0.877
	KTx	52	44.38	6.278		
spr_dem	LTx	44	27.25	8.461	−0.607	0.545
	KTx	52	28.31	8.551		
spr_aut	LTx	44	38.02	4.593	0.199	0.842
	KTx	52	37.81	5.767		
spr_inc	LTx	44	20.05	7.886	−0.906	0.367
	KTx	52	21.52	7.989		
spr_prot	LTx	44	28.86	10.197	−1.324	0.189
	KTx	52	31.44	8.89		

**Table 2:** Pearson’s correlations between the overall resilience score, parental attitudes and the regularity of taking medications.

	spr_acc	spr_dem	spr_aut	spr_inc	spr_prot	Morisky MMAS-8
SPP_overall	0.34 **	−0.03	0.27 **	−0.00	0.00	0.26 **
spr_acc		−0.07	0.20 *	−0.30 **	0.33 **	0.23 *
spr_dem			−0.19 *	0.68 **	0.56 **	0.07
spr_aut				−0.12	−0.16	−0.03
spr_inc					0.35 **	0.05
spr_prot						0.27 **

* *p* < 0.05; ** *p* < 0.01.

**Table 3:** Clinical predictors of regularity of drug intake.

Model		β	t	Significance	The Significance Model	R^2^	ΔR^2^	The Significance of the Change
1	SPP_overall	0.256	2.550	0.012	F(1.94) = 6.502. *p* = 0.12	0.065	0.065	0.012
2	SPR_acc	0.069	0.527	0.600	F(6.94) = 2.622. *p* = 0.22	0.152	0.086	0.123
	SPR_dem	−0.105	−0.683	0.497				
	SPR_aut	−0.083	−0.784	0.435				
	SPR_inc	0.034	0.232	0.817				
	SPR_prot	0.281	2.053	0.043				
3	SPP_x_SPR_acc	0.412	1.779	0.047	F(11.94) = 2.545. *p* = 0.08	0.252	0.101	0.050
	SPP_x_SPR_dem	−0.167	−1.021	0.310				
	SPP_x_SPR_aut	0.056	0.328	0.744				
	SPP_x_SPR_inc	0.247	1.619	0.109				
	SPP_x_SPR_prot	−0.033	−0.177	0.860				

## Data Availability

The data presented in this study are available on request from the corresponding author.
